# Integrated Transcriptomic Analysis Provided Diagnostic and Pathophysiological Insights for Epilepsy

**DOI:** 10.1155/jimr/5925485

**Published:** 2025-07-15

**Authors:** Shuang Li, Zhigang Wang, Yake Zheng, Yunqing Ma, Zhi Huang, Yajun Lian

**Affiliations:** ^1^Department of Neurology, The First Affiliated Hospital of Zhengzhou University, Zhengzhou 450052, China; ^2^Department of General Medicine Ward 3, The First Affiliated Hospital of Zhengzhou University, Zhengzhou 450052, China; ^3^Department of Stroke ICU, The First Affiliated Hospital of Zhengzhou University, Zhengzhou 450052, China

**Keywords:** diagnostic model, enrichment analysis, epilepsy, hub genes, T cell function

## Abstract

**Background:** Epilepsy is a common neurological disorder involving multiple genes and molecular pathways. Study of differentially expressed genes (DEGs) and hub genes related to epilepsy can help reveal the pathophysiologic basis and improve potential diagnostic and therapeutic strategies.

**Methods:** Transcriptome data of two epilepsy datasets (GSE143272 and GSE32534) and single-cell sequencing data (GSE201048) were collected from the Gene Expression Omnibus (GEO) database. Differential expression analysis was performed using Limma R package, and the hub genes were identified and analyzed utilizing STRING database and Cytoscape software. The clusterProfiler R package was used to perform gene function enrichment analysis and an epilepsy diagnostic model was constructed with the hub genes. The model performance was assessed according to receiver operating characteristic (ROC) curves.

**Results:** Multiple DEGs linked to epilepsy were identified and 20 common DEGs between the two datasets were revealed. Eleven hub genes closely associated with epilepsy were identified by protein–protein interaction (PPI) network analysis. CD3D, CD3G, CTSW, and JCHAIN were consistently expressed in the GSE143272 and GSE32534 datasets and all showed a low expression in epilepsy samples. In particular, the diagnostic model developed with the four genes demonstrated a strong discriminatory ability in both datasets (all area under curve (AUC) > 0.7). Functional enrichment and single-cell analysis revealed that these key genes were closely related to T cell function, suggesting that they may play an important role in the immune regulation of epilepsy.

**Conclusion:** This study successfully identified four key genes linked to epilepsy, contributing to the molecular diagnosis of epilepsy.

## 1. Introduction

Epilepsy is a neurological disorder caused by various etiological factors that lead to the abnormal synchronization of multiple neurons in the brain. Patients with epilepsy show episodic, repetitive, stereotyped, and transient seizures [1, 2]. According to the epidemiological studies, over 70 million patients worldwide are affected by epilepsy, with an increasing trend [3]. Currently, epilepsy is mainly controlled by taking oral antiepileptic drugs (AEDs) properly and regularly. About 60%–70% of epilepsy patients achieve symptom relief with AEDs and around 30% develop refractory epilepsy [4, 5]. At present, epilepsy pathogenesis is complex and remains incompletely understood [6]. Genetic mutations, inflammation, and immune factors are recognized as key contributing factors. Accumulating evidence showed that the development of epilepsy is inextricably linked to gene mutations. *SCN1A* gene mutation is an important gene linked to seizures [7]. SCN1A consists of 26 coding exons and its mutation may disrupt gamma-aminobutyric acid function and sodium channel dysfunction and in turn cause neuronal hyperexcitability, thereby contributing to the development of epilepsy [6, 8]. In addition, interleukin-1β can induce Src kinase-mediated tyrosine phosphorylation of the NR2B subunit of the N-methylaspartate receptor through enhancing N-methylaspartate receptor-mediated Ca^2+^ influx to further activate neuronal excitability and cause epilepsy seizures [9].

Previous research found that inflammation may be an important factor resulting in seizures [10]. Patients with epilepsy have an overactive immune system, which may further contribute to a higher frequency of autoimmune seizures [11, 12]. The study concluded that patients with T cell-mediated autoimmune encephalitis are more likely to develop epilepsy [13]. Helmstaedter et al. [14] demonstrated that cognition in patients with epilepsy in the autoantibody-positive group is correlated with T and B cell activity, CD4^+^ T cells and CD8^+^ T cells in the blood, and CD4^+^ T cells in the cerebrospinal fluid. Follicular helper T cell subsets is also linked to the occurrence of seizures [15]. These findings point to the need to further explore the mechanisms of T cell action in epilepsy.

This study identified hub genes of epilepsy based on computational analysis, gene network, and coexpression network. Functional enrichment analysis showed that the hub genes were closely associated with T cell function. Single-cell analysis of the association between the hub genes and T cells determined four genes that may crucially affect T cell function in epilepsy. The present findings provided a scientific basis for the pathogenesis of epilepsy.

## 2. Materials and Methods

### 2.1. Analysis Data Acquisition

Transcriptome microarray sequencing data of two epilepsy research projects (GSE143272 and GSE32534) were collected from the Gene Expression Omnibus (GEO; https://www.ncbi.nlm.nih.gov/geo/) database. The probes were converted to symbol according to the annotation file. Finally, GSE143272 dataset contained 85 samples (51 control samples and 34 epilepsy samples). GSE32534 dataset contained five control samples and five epilepsy samples. In addition, we obtained single-cell sequencing data from GSE201048 dataset, an epilepsy single-cell research dataset, from the GEO database, which contained 11 epilepsy samples. The library building platform was 10x Genomics and the sequencing platform was Illumina NovaSeq 500.

### 2.2. Identification of Differentially Expressed Genes (DEGs) in the Epilepsy Samples

The DEGs in the epilepsy samples from GSE143272 and GSE32534 datasets were filtered under the criteria of *p* < 0.05 and |log_2_ fold change (FC)| > log_2_ (1.5) using R package limma, with normal samples as the control group [16, 17]. Overlapping DEGs were served as the candidate hub genes for subsequent identification of hub genes in epilepsy [18].

### 2.3. Protein–Protein Interaction (PPI) Network for Screening Hub Genes

To identify hub genes in epilepsy, the overlapping DEGs were imported into the STRING database (https://cn.string-db.org/) under minimal required interaction score of 0.4, followed by the development of a PPI network [19]. The obtained PPI network was imported into Cytoscape software. The top 10 genes screened by the five algorithms (the built-in maximal clique centrality (MCC), maximum neighborhood component (MNC), closeness, edge percolated component (EPC), and degree algorithms of CytoHubba plug-in) were intersected and the resulting DEGs were considered as the hub genes in epilepsy [20]. Finally, these hub genes were imported into the GeneMANIA database and a gene coexpression network was established by recognizing the coexpressed genes [21].

### 2.4. Gene Function Enrichment Analysis

The biological processes (BPs) and pathways involved in the hub genes were explored using Gene Ontology (GO, in the three terms of molecular functions (MFs), cellular components (CCs), and BPs) and Kyoto Encyclopedia of Genes and Genomes (KEGG) analysis with the R package clusterProfiler [22, 23]. Terms with *p* < 0.05 and FDR < 0.05 were considered to be statistically significant.

### 2.5. Construction a Genetic Diagnostic Model for Epilepsy

The expressions of all the hub genes in GSE143272 and GSE32534 datasets were analyzed to select the genes with consistent expression trend to construct a gene diagnostic model for epilepsy. To validate the diagnostic performance of the model, the receiver operating characteristic (ROC) curve of the hub genes was plotted for GSE143272 and GSE32534 using the R package ROCR and the area under curve (AUC) values were calculated [24].

### 2.6. Regulatory Networks Involving the Hub Genes

Transcription factors (TFs) control gene expression at the post-transcriptional stage by interacting with hub genes. The regulatory role of the TFs that interacted with the hub genes was analyzed based on the TFs retrieved from the ChIPBase (https://rnasysu.com/chipbase3/) database, and the mRNA-TF regulatory network was visualized by Cytoscape software. In addition, to comprehensively analyze the relationship between the hub genes and miRNAs, miRNAs related to the hub genes were collected from the miRDB database and a mRNA–miRNA regulatory network [20] was visualized by Cytoscape software.

### 2.7. Analysis on the Epilepsy Single-Cell Data

We used the Read10X function of the R package Seurat to read the data, retaining cells with a mitochondrial gene proportion of <10% and a gene count between 200 and 6000 [25]. Data were normalized by the SCTransform function, followed by performing principal component analysis (PCA) with the RunPCA function. The harmony package was used to remove the batch effect between different samples [26]. Here, we utilized the first 20 PCs for UMAP downscaling. The FindNeighbors and FindClusters functions were applied to cluster cell subpopulations (resolution = 0.1). Finally, we annotated each cell type with the marker genes provided by the CellMarker 2.0 database [27, 28].

### 2.8. Statistical Analysis

The raw data were preprocessed in the R statistical environment (version 4.2.1). To identify significant differences between two independent cohorts, Student's *t* test or Wilcoxon rank-sum test was employed depending on the data distribution. Additionally, the Kruskal–Wallis test was used, followed by Dunn's test for post hoc multiple comparisons among different groups. A *p*-value of <0.05 denoted statistical significance.

## 3. Results

### 3.1. Functions of the DEGs in Epilepsy

**Figure 1:**
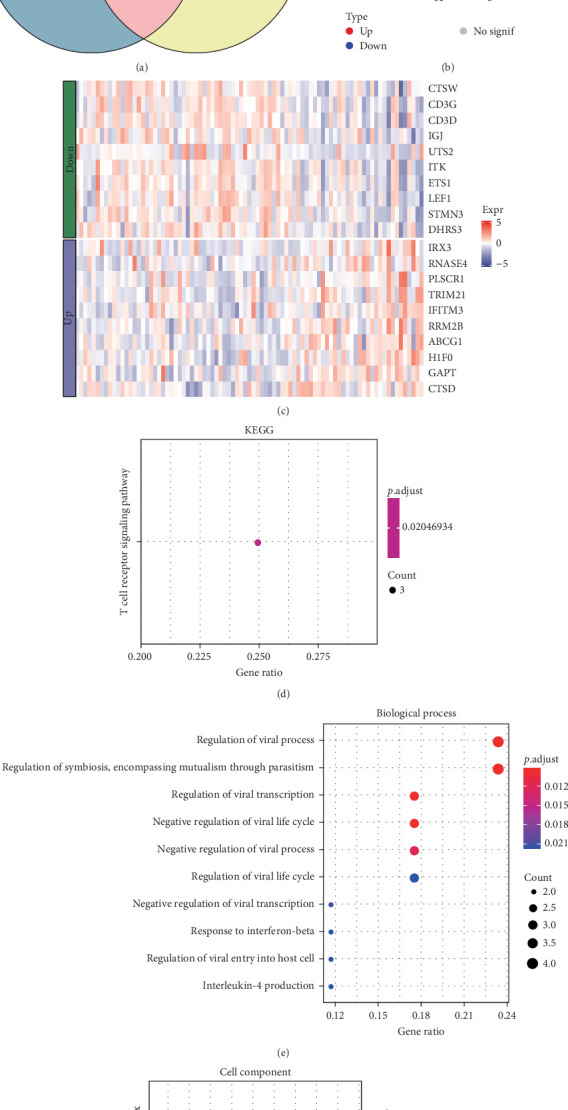
Functions of DEGs in epilepsy. (A) Wayne diagram showing DEGs in the GSE143272 and GSE32534 datasets and overlapping DEGs in both. (B) Volcano plot of up- and downregulated DEGs in the epilepsy sample in the GSE143272 dataset. (C) Heatmap showing the expression levels of the 20 overlapping DEGs in the GSE143272 dataset. (D) KEGG pathway involved in overlapping DEGs. (E) Biological Process involved in overlapping DEGs. (F) Cell component involved in overlapping DEGs.

A total of 20 overlapping DEGs were present in the intersection between 335 DEGs in GSE143272 dataset and 323 DEGs in GSE32534 dataset (Figure 1A). In the GSE143272 dataset, 180 upregulated DEGs and 155 downregulated DEGs were found in epilepsy samples (Figure 1B). The expressions of 20 overlapping DEGs in epilepsy samples between the two datasets were visualized into a gene expression heatmap (Figure 1C). Finally, GO and KEGG enrichment analysis on the 20 DEGs showed that these genes were enriched in T cell receptor signaling pathway (Figure 1D). In BP term, these DEGs were mainly involved in encompassing mutualism through parasitism, negative regulation of viral life cycle, regulation of symbiosis, regulation of viral process, regulation of viral transcription, and negative regulation of viral process (Figure 1E). In CC term, the proteins translated by overlapping DEGs were primarily constituent proteins of the T cell receptor complex (Figure 1F).

### 3.2. PPI Network Identification of the Hub Genes in Epilepsy

**Figure 2:**
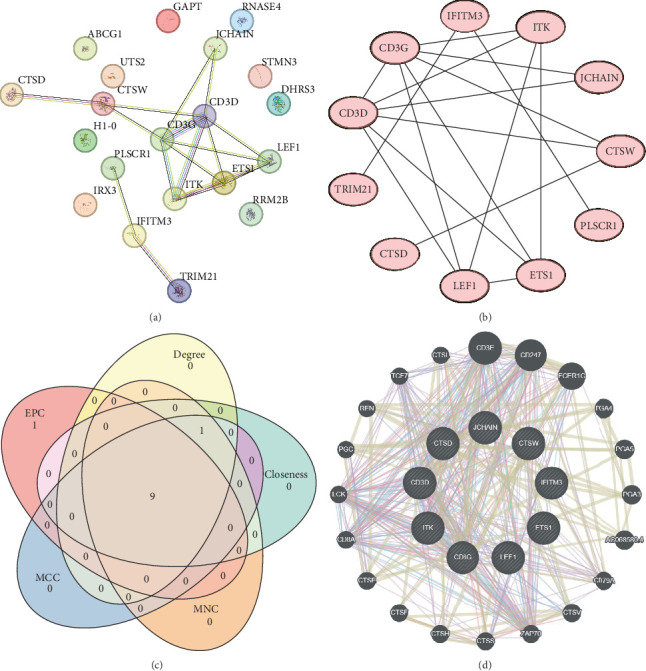
PPI network identification of hub genes in epilepsy. (A) PPI network of 20 overlapping genes. (B) PPI network of 11 potential genes with minimum required interaction score above 0.4. (C) Wayne diagram showing the top 10 genes with nine overlapping hub genes identified by each of the five algorithms in Cytoscape software. (D) Coexpression network of nine-hub genes identified in GeneMANIA database.

A PPI network of the 20 overlapping DEGs was developed by the STRING database, and we identified 11 potential hub genes in the network, including IFITM3, ITK, JCHAIN, CTSW, PLSCR1, ETS1, LEF1, CTSD, TRIM21, CD3D, and CD3G (Figure 2A,B). Feature selection was performed using MCC, MNC, Closeness, EPC, and degree algorithms with the Cytoscape software, and the top 10 genes identified by each algorithm were intersected to obtain nine genes, which we considered as the hub genes in epilepsy (Figure 2C). Finally, we constructed a gene coexpression network of the nine hub genes, and there were 20 genes showing coexpression with the nine hub genes (Figure 2D).

### 3.3. A Diagnostic Model Was Established Using the Hub Genes for Epilepsy

**Figure 3:**
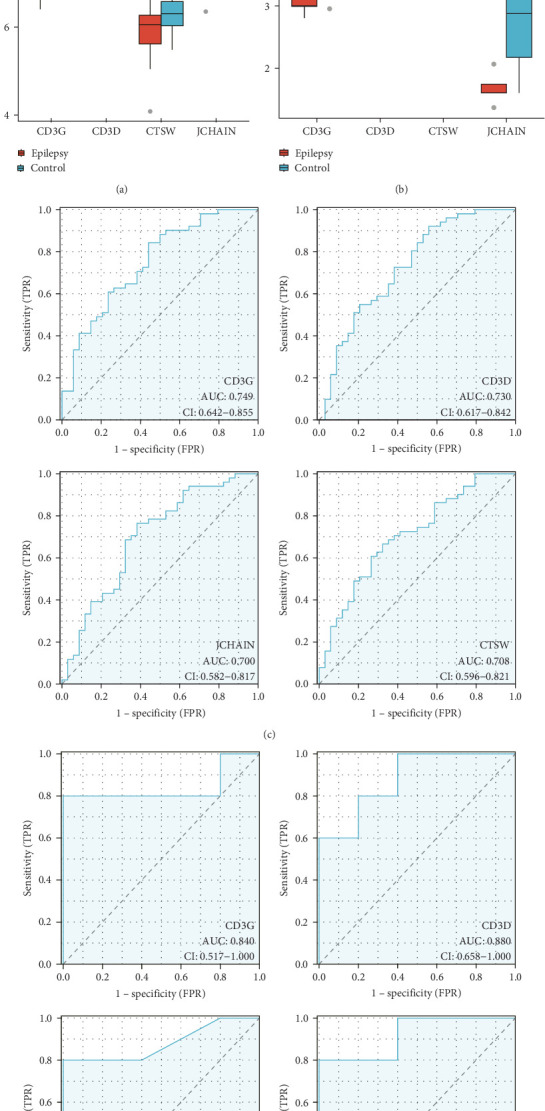
Hub genes composition of epilepsy diagnostic model. (A) Expression levels of CD3D, CD3G, CTSW, and JCHAIN in normal and epilepsy samples in the GSE143272 dataset. (B) Expression levels of CD3D, CD3G, CTSW, and JCHAIN in normal and epilepsy samples in the GSE32534 dataset. (C) ROC curves of CD3D, CD3G, CTSW, and JCHAIN in diagnostic epilepsy samples in GSE143272 dataset. (D) ROC curves of CD3D, CD3G, CTSW, and JCHAIN in diagnostic epilepsy samples in GSE32534 dataset. *⁣*^*∗*^ indicates *p* < 0.05, *⁣*^*∗∗*^ indicates *p* < 0.01, and *⁣*^*∗∗∗*^ indicates *p* < 0.001.

The expressions of nine hub genes were visualized in the GSE143272 and GSE32534 datasets. Among them, we observed that the levels of CD3D, CD3G, CTSW, and JCHAIN were all significantly downregulated in epilepsy samples in the GSE143272 and GSE32534 datasets (Figure 3A,B). A diagnostic model of epilepsy was constructed utilizing the four genes, and the model robustness was tested in the two datasets. Specifically, CD3D (AUC = 0.730, 95% CI = 0.617–0.842), CD3G (AUC = 0.749, 95% CI = 0.642–0.855), CTSW (AUC = 0.708, 95% CI = 0.596−0.821), and JCHAIN (AUC = 0.700, 95% CI = 0.582–0.817) all had an AUC value higher than 0.70 in the dataset GSE143272, suggesting a strong diagnostic performance of the four genes (Figure 3C). In GSE32534, CD3D (AUC = 0.880, 95% CI = 0.658–1.000), CD3G (AUC = 0.840, 95% CI = 0.517–1.000), CTSW (AUC = 0.920, 95% CI = 0.736−1.000), and JCHAIN (AUC = 0.880, 95% CI = 0.634–1.000) all had an AUC value higher than 0.80, further indicating that the diagnostic model could effectively differentiate epilepsy patients (Figure 3D).

### 3.4. The Hub Genes Were Linked to the Function of T Cells

**Figure 4:**
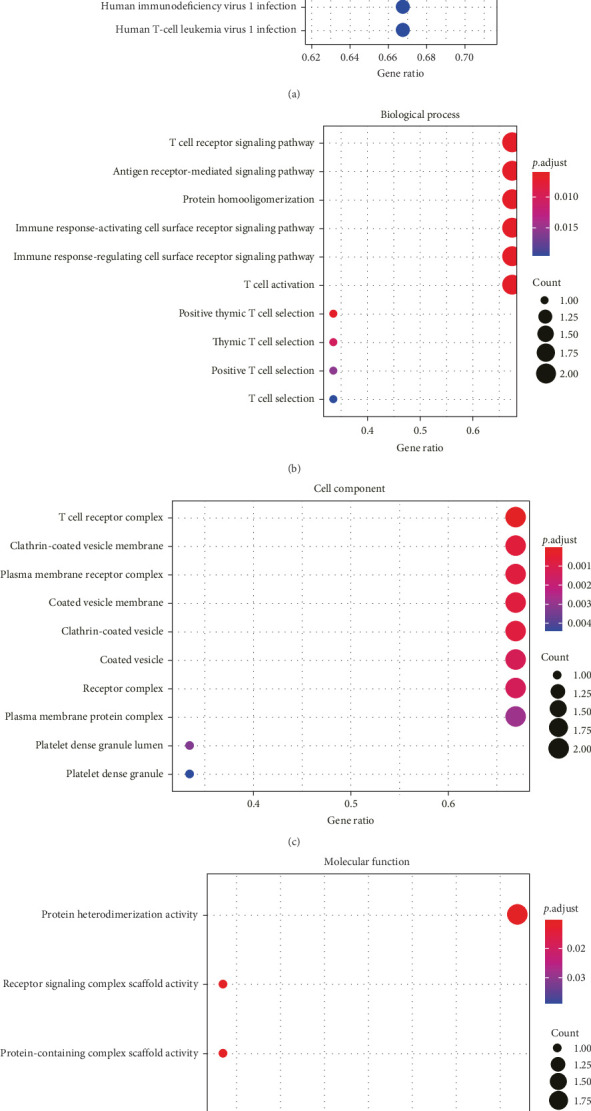
Hub genes are associated with T cell function. (A) Bubble diagram of KEGG pathway involved in hub genes. (B) Bubble diagram of GO_biological process terms involved in hub genes. (C) Bubble diagram of GO_cell component terms involved in hub genes. (D) Bubble map of GO_molecular function terms involved in hub genes.

All the hub genes obtained in the study were subjected to KEGG and GO functional enrichment analysis. We found that these genes were involved in Th1 and Th2 cell differentiation, PD-L1 expression and PD-1 checkpoint pathway in cancer, hematopoietic cell lineage, T cell receptor signaling pathway, Chagas disease (American trypanosomiasis), and Th17 cell differentiation pathway (Figure 4A). In BP term, these hub genes were closely correlated with T cell function, including positive thymic T cell selection, T cell selection, thymic T cell selection, T cell receptor signaling pathway, positive T cell selection, and T cell activation (Figure 4B). In CC term, the hub genes translated proteins that may be the structural components of the T cell receptor complex, clathrin-coated vesicle membrane, plasma membrane receptor complex, coated vesicle membrane, and clathrin-coated vesicle (Figure 4C). Finally, in MF term, the hub genes were functionally linked to protein heterodimerization activity, protein-containing complex scaffold activity, receptor signaling complex scaffold activity, and cysteine-type peptidase activity (Figure 4D).

### 3.5. The Network of miRNA–Hub Gene–TFs

**Figure 5:**
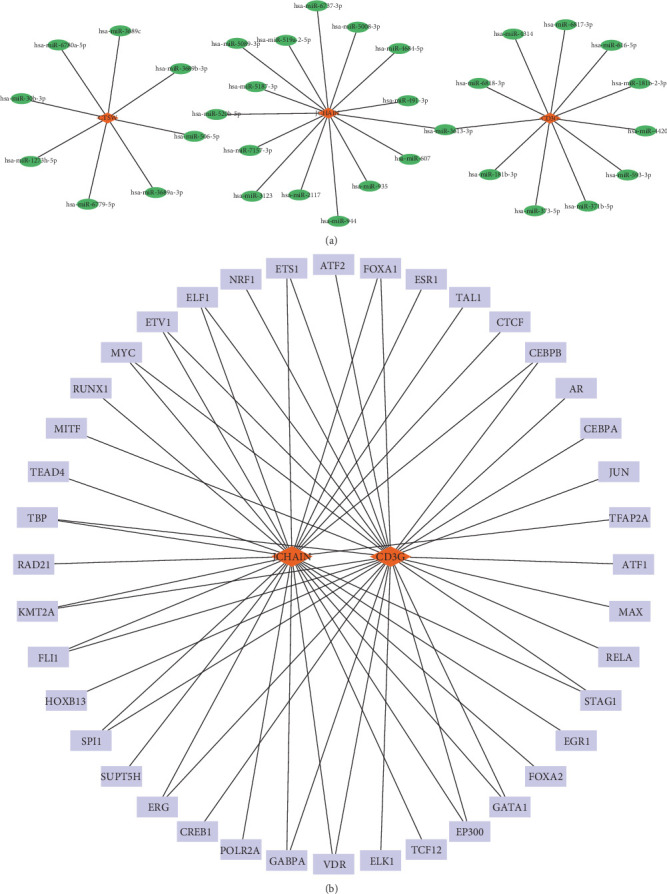
miRNA-hub genes-TFs network. (A) The miRNA–mRNA network regulated by CTSW, JCHAIN, and CD3G. (B) The mRNA-TFs network regulated by CTSW, JCHAIN, and CD3G.

To reveal the multilevel regulatory mechanisms of the key genes in epilepsy at posttranscriptional and transcriptional levels, this study constructed a miRNA–hub gene–TF regulatory network to provide a theoretical basis for their functions and potential intervention targets. In miRDB database, miRNAs related to hub genes were selected under a target score higher than 80 as the screening condition and CTSW, JCHAIN, and CD3G met the conditions. Here, eight miRNAs had regulatory relationships with CTSW, 15 miRNAs had regulatory relationships with JCHAIN, and 11 miRNAs had regulatory relationships with CD3G (Figure 5A). Meanwhile, TFs related to at least five downstream targets mentioned above were screened as potentially having a regulatory relationships with the hub genes. Here, JCHAIN and CD3G are coregulated by 40 TFs (Figure 5B).

### 3.6. Expression Levels of the Hub Genes in Immune Cells

**Figure 6:**
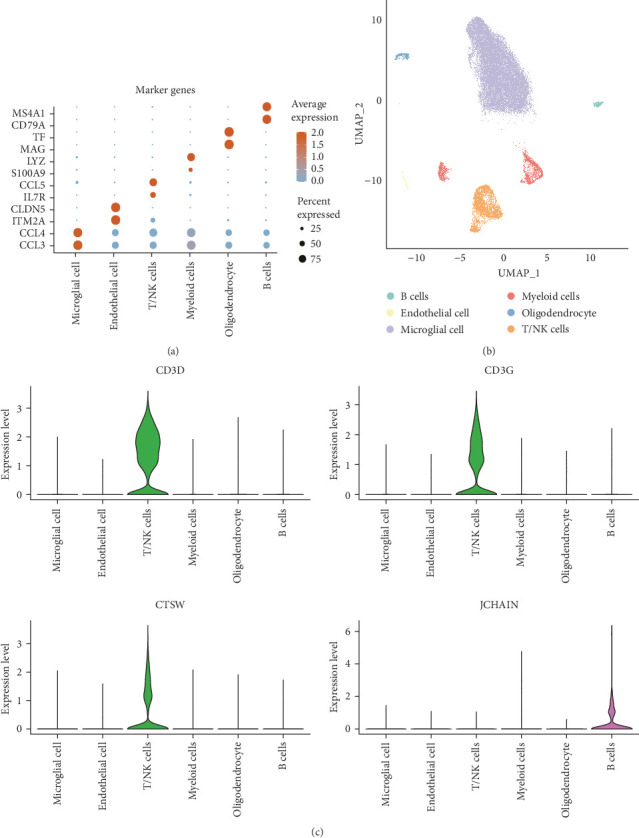
Expression levels of hub genes in immune cells. (A) Expression levels of marker genes in cell subpopulations. (B) Single-cell mapping of cell subpopulations. (C) Expression levels of hub genes in six cell subpopulations.

A total of 11 epilepsy samples from single-cell dataset GSE201048 were used for data analysis. After cell filtering, SCT normalization, UMAP downscaling, and unsupervised clustering, data of 58,385 cells were considered analytically significant. Based on the expression levels of the marker genes, six cell subpopulations were classified as follows: microglial cells (marked with CCL3 and CCL4), endothelial cells (marked with ITM2A and CLDN5), T cells and natural killer (NK) cells (T/NK cells, marked with IL7R and CCL5), myeloid cells (marked with S100A9 and LYZ), oligodendrocyte (marked with MAG and TF), and B cells (marked with CD79A and MS4A1; Figure 6A,B). In addition, we analyzed the expressions of the four hub genes in immune cells. CD3D and CD3G were high-expressed in T/NK cells. This suggested the importance of T cell receptor signaling in epilepsy and the association between neuroinflammation and immune regulation in the disease. CTSW was also high-expressed in T/NK cells, further supporting the critical role of T cells in epilepsy, especially in cytotoxic activities. JCHAIN was high-expressed in B cells, suggesting that B cells and antibody-mediated immune responses may influence the progression and severity of epilepsy (Figure 6C).

## 4. Discussion

Epilepsy is a neurological condition that encompasses both hereditary and acquired disorders and influences at least 46 million patients all over the world [29]. Transcriptomic analysis has been widely applied in screening markers for epilepsy, providing new insights into the disease pathogenesis and effective targets [30]. This study was the first to systematically identify key genes closely related to T cell function for epilepsy by integrating transcriptome and single-cell sequencing data, and a molecular model with a high diagnostic efficacy was developed to reveal the potential mechanism of immune-related genes in epilepsy. A total of nine hub genes were screened in this study, in particular, four hub genes (CD3D, CD3G, CTSW, and JCHAIN) could effectively distinguish epilepsy samples. Functional analysis showed that the four hub genes were significantly correlated with T cell functional activity. CD3D is directly involved in T cell development, cell migration, and activation signaling pathways and is associated with propionate treatment resistance in epilepsy [31]. Currently, functional analysis of CD3D in epilepsy is limited, but CD3D has been found to be associated with immune checkpoints [32]. CD3D mutations in T cells cause destructive immunity innate errors [33], while CD3G deletion leads to T cell immunodeficiency [34]. In our research, both CD3D and CD3G were downregulated in epilepsy samples. Previous study reported that severe immunodeficient phenotype of epilepsy with deletion of CD3D and CD3G may cause defective T cell development and lead to the early onset of the disease [10–12]. Hence, we hypothesized that suppression of CD3D and CD3G expression limited T cell immune function and that impaired autoimmune function exacerbated the progression of epilepsy. CTSW may play a crucial role in epilepsy resistance and is a hub gene in a diagnostic model of drug-resistant epilepsy [35]. Peripheral regulatory T cells (pTregs) serve as a key T cell type in mucosal immune tolerance and anti-inflammatory responses, and loss of CTSW expression increases pTregs and promotes immune responses in intestinal inflammation [36]. As shown in our findings, CTSW expression was also downregulated in epilepsy samples. JCHAIN has been found to be associated with immunotherapeutic response [37]. JCHAIN-derived TCR therapy is a promising treatment for multiple myeloma [38]. These studies also validated our results that the four hub genes were closely correlated with T cell function.

According to the AUC values, the epilepsy diagnostic model constructed based on the four hub genes also showed excellent diagnostic performance in both GSE143272 and GSE32534 datasets. Accurate diagnosis and timely treatment are critical in epilepsy management to ensure optimal therapeutic outcomes. Early identification of epilepsy is particularly essential given the diverse etiologies of the disease, including stroke, infection, traumatic brain injury, metabolic disorders, autoimmune diseases, and even genetic factors [39]. The present model can identify potential epilepsy patients based on gene expressions together with clinical assessments, neuroimaging and neuropathology as auxiliary methods.

It should be noted that there were several limitations in this study. First, the transcriptome datasets used had a small sample size and differences in sequencing platforms and experimental conditions, which may affect the reliability of the genes identified and the generalization ability of the model. Therefore, we will further integrate larger, multiplatform and multicenter epilepsy samples in combination with clinical samples for external validation to improve the robustness of the present results. Second, the specific mechanism of the roles of CD3D, CD3G, CTSW, and JCHAIN in the development of epilepsy has not been verified by cell or animal experiments. Thus, the biological functions and pathogenic mechanisms of the key genes should be systematically evaluated together with immune cell function experiments (e.g., T cell proliferation, activation, and cytotoxicity assay) and animal models. In addition, human T cells, primary T cells, or brain-like organ models will be used to simulate the interaction environment between immune cells and neural cells so as to explore the roles of the key genes in neuroimmune interaction. Finally, this study did not provide an in-depth analysis of the specific subtypes of epilepsy patients, which may cause certain generalization bias to the results. In the future, different expression patterns and functional heterogeneity of the key genes in different epilepsy subtypes will be analyzed using the cohorts with complete clinical information to improve the accuracy and applicability of our results. In general, further studies are encouraged to expand the functional landscape of the key immune-related genes in epilepsy, particularly focusing on their dynamic expression patterns across different disease stages, pathological subtypes, and therapeutic response contexts. In addition, integrating our molecular diagnostic model with multimodal clinical data, such as neuroimaging and electroencephalography, may contribute to a more individualized diagnostic treatment for epilepsy, which could provide a theoretical foundation and technical support for precision medicine.

## 5. Conclusion

Integrated transcriptomic and single-cell sequencing analyses identified CD3D, CD3G, CTSW, and JCHAIN as the key epilepsy genes that were also closely related to T cell function. A molecular model with a high diagnostic efficacy was constructed using the four genes. Functional enrichment and cellular subpopulation analysis showed that these genes may be involved in epileptogenesis and development of epilepsy by influencing T cell-mediated immunomodulation. In conclusion, this study discovered novel biomarkers for the molecular diagnosis of epilepsy, providing a theoretical foundation for its immunopathological mechanisms.

## Data Availability

The datasets generated and/or analyzed during the current study are available in the GSE143272 repository (https://www.ncbi.nlm.nih.gov/geo/query/acc.cgi?acc=GSE143272), GSE32534 repository (https://www.ncbi.nlm.nih.gov/geo/query/acc.cgi?acc=GSE32534), and GSE201048 repositories (https://www.ncbi.nlm.nih.gov/geo/query/acc.cgi?acc=GSE201048).

## Nomenclature

GEO: Gene Expression Omnibus
DEGs: Differentially expressed genes
GO: Gene Ontology
KEGG: Kyoto Encyclopedia of Genes and Genomes
BPs: Biological processes
CCs: Cellular components
MFs: Molecular functions
ROC curve: Receiver operating characteristic curve
AUC: Area under curve
TFs: Transcription factors
pTregs: Peripheral regulatory T cells.
